# Sex-specific associations of cardiovascular risk factors and coronary plaque composition for hemodynamically significant coronary artery stenosis: a coronary computed tomography angiography study

**DOI:** 10.1186/s12872-023-03438-x

**Published:** 2023-08-27

**Authors:** Mengshan Wu, Jintang Feng, Zhang Zhang, Ningnannan Zhang, Fan Yang, Ruijun Li, Yueqi Men, Dong Li

**Affiliations:** 1 Department of radiology, Tianjin Medical University General Hospital, 154 Anshan Road, Tianjin, China; 2 Department of radiology, Tianjin Hospital, 406 Jiefang South Road, Tianjin, China

**Keywords:** Coronary artery disease, Coronary computed tomography angiography, Sex, Heart disease risk factors, Coronary stenosis

## Abstract

**Background:**

It has been reported that there are sex differences in plaque composition and hemodynamically significant stenosis. This study aimed to explore the impact of sex on cardiovascular risk factors for specific plaque compositions and hemodynamically significant stenosis.

**Methods:**

Data regarding demographics and cardiovascular risk factors were collected. Hemodynamically significant stenosis was identified by a computed tomography-derived fractional flow reserve of ≤ 0.8. Associations among cardiovascular risk factors, plaque composition, and hemodynamically significant stenosis were assessed using a multivariate binary logistic regression analysis across sexes. The discriminating capacity of diverse plaque components for hemodynamically significant stenosis was assessed by area under the receiver-operating characteristics curve with 95% confidence intervals.

**Results:**

A total of 1164 patients (489 men and 675 women) were included. For men, hyperlipidemia and cigarette smoking were risk factors for each plaque component (all P < 0.05), and diabetes mellitus also predicted fibrotic components (P < 0.05). For women, risk factors for each plaque component were hypertension and diabetes mellitus (all P < 0.01). Nonetheless, hyperlipidemia (P < 0.05) was a specific risk factor for non-calcified components. Calcified components combined with fibrotic components showed superior discrimination of hemodynamically significant stenosis in men and calcified components alone in women (all P < 0.01). Hypertension (P < 0.01) was a risk factor for hemodynamically significant stenosis in women. In contrast, diabetes, hyperlipidemia, and cigarette smoking were risk factors for hemodynamically significant stenosis in men (all P < 0.05).

**Conclusions:**

In men, hemodynamically significant stenosis was predicted by a combination of calcified and fibrotic components with multiple risk factors. In women, hemodynamically significant stenosis was predicted by calcified components caused by a single risk factor. It might be a key point to improve prognosis by more precise risk management between men and women, which needs to be proved by further prospective trials.

**Supplementary Information:**

The online version contains supplementary material available at 10.1186/s12872-023-03438-x.

## Background

Coronary artery disease (CAD) is the leading cause of death for men and women [1]. Previous studies have found sex-related differences in the incidence of, severity of, and prognosis for CAD [2, 3]. Although men present earlier in life, have higher clinical cardiovascular risk assessment scores, and have a significantly higher incidence of obstructive CAD, women have a worse prognosis [3, 4]. It is unclear how cardiovascular risk factors affect CAD in men and women.

The culprit plaque maybe a key risk factor for ischemia. In healthy individuals, the common epicardial coronary artery shows slight resistance to myocardial blood flow. With increasing stenosis severity caused by plaque progression, resistance to blood flow begins to increase, eventually leading to insufficient blood supply to the corresponding myocardium. Nonetheless, there are crucial questions regarding anatomic or functional mismatch. Real-world myocardial ischemia is directly caused by the obstruction of blood flow rather than coronary stenosis [5, 6]. Therefore, whether there are sex differences in the cardiovascular risk factors for hemodynamically significant stenosis is unclear.

The diverse plaque components have varying clinical significance. A higher calcified component volume indicates a more stable and mature plaque, which can contribute to obstructive CAD in the chronic phase. Conversely, a higher non-calcified component volume, particularly lipid-rich components, signifies an active and unstable plaque. Acute thrombosis caused by lipid-rich plaque rupture results in acute coronary syndrome, contributing to major adverse cardiovascular events [7]. Coronary computed tomography angiography (CCTA), as the first-line noninvasive clinical examination method, can visualize coronary plaques, including plaque burden, location, composition, and stenosis severity, which provides accurate diagnostic information and a high negative predictive value [8, 9]. Furthermore, the computed tomography–derived fractional flow reserve (FFR_CT_) has been demonstrated to enhance the discrimination of hemodynamically significant stenosis by simulating hemodynamic information from the calculation of the vessel lumen volume shape and is better than CCTA alone or other noninvasive tests [10, 11].

This study aimed to determine any sex differences in associations among cardiovascular risk factors, plaque composition obtained using CCTA, and hemodynamically significant stenosis identified using the FFR_CT_.

## Methods

### Study design and patient population

**Fig. 1:**
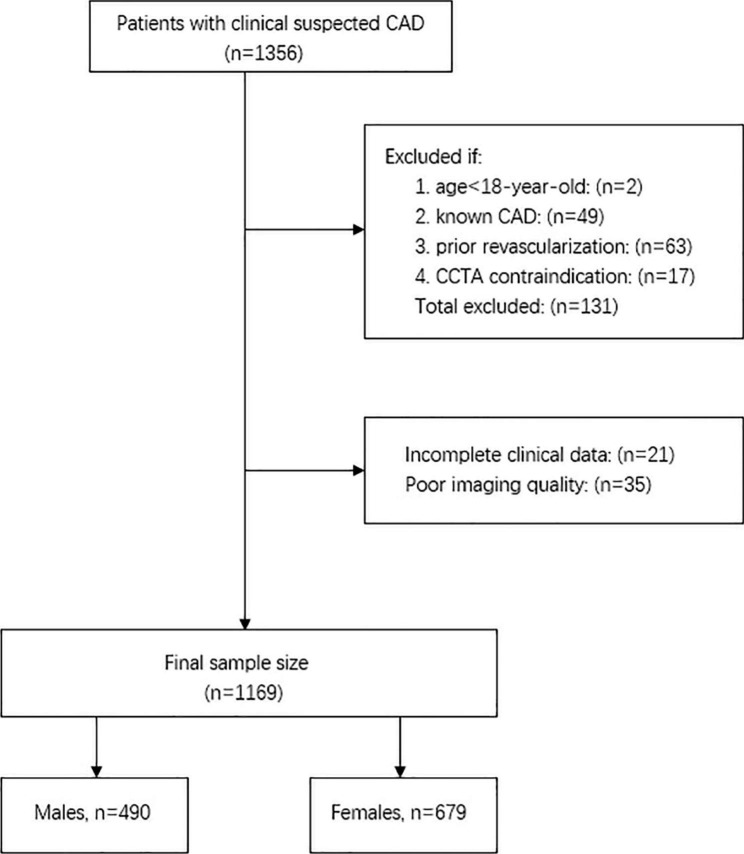
Flow chart of study inclusion and exclusion

This prospective observational study was approved by the Institutional Review Board, and all patients provided written informed consent. Outpatients with suspected clinical CAD who underwent CCTA were continuously enrolled between April 2018 and June 2019. The exclusion criteria for this study were age < 18 years, known CAD or prior revascularization (myocardial infarction, percutaneous coronary intervention, or coronary artery bypass graft surgery), and contraindications for CCTA (Fig. 1).

### Individual demographics and clinical traditional risk factor assessment

We collected data regarding individual demographics and cardiovascular risk factors. The demographic data included sex, age, height, weight, and exercise status. Body mass index (BMI) was calculated as weight (kg) divided by height in meters squared (m^2^). Exercise status was categorized into the following three grades: 0 points, no exercise; 1 point, exercise performed once or twice per week; and 2 points, exercise performed three or more times per week. Only one person received menopausal hormone therapy.

Information on hypertension, hyperlipidemia, diabetes mellitus, cerebrovascular diseases, family history of premature CAD (the incidence of CAD in first-degree male and female relatives at ages < 55 years and < 65 years, respectively) [12], and smoking status was systematically acquired. Hypertension was defined as a self-reported history of hypertension and/or the use of antihypertensive medication or blood pressure ≥ 140/90 mmHg. Hyperlipidemia was defined as serum total cholesterol ≥ 5.20 mmol/L, triglycerides ≥ 1.7 mmol/L, high-density lipoprotein cholesterol ≤ 1.04 mmol/L, and low-density lipoprotein cholesterol ≥ 3.61 mmol/L and/or the use of statins. Diabetes was defined as a self-reported history of diabetes and/or receiving anti-diabetic treatment, fasting plasma glucose level ≥ 7.0 mmol/L, or 2-hour postprandial plasma glucose level ≥ 11.1 mmol/L. Cerebrovascular disease was defined as a self-reported history of the disease. Smoking status was categorized into the following three groups: 0 point, never smoked; 1 point, quit smoking for ≥ 1 year; and 2 points, current smoking or quit smoking for < 1 year.

Antihypertensive agents were classified as diuretics (1.25 − 50 mg/day), β-blockers (5 − 160 mg/day,), α-blockers (1 − 20 mg/day), calcium channel blockers (2.5 − 120 mg/day), angiotensin-converting enzyme inhibitors (5 − 150 mg /day), angiotensin II receptor blockers (8 − 320 mg/day), centrally acting drugs (150 − 300 mg/day) and vasodilators (0.1 − 2 mg/day). Oral hypoglycemic agents were grouped into six classes, including biguanide, e.g., metformin (500 − 2000 mg/day), sodium-glucose cotransporter-2 inhibitors (10 − 25 mg/day), glucagon-like peptide 1 receptor agonists (0.3 − 1.8 mg/day), dipeptidyl peptidase 4 inhibitors (5 − 100 mg/day), thiazolidinediones (4 − 45 mg/day), and sulfonylureas (2.5 − 180 mg/day). Insulin use was recommended at 0.1 − 0.3 U/kg/day.

### Coronary computed tomography angiography acquisition

CCTA examinations were performed using a third-generation, 64-row multidetector, dual-source CT scanner (SOMATOM Force CT; SIEMENS, Munich, Germany) with a detector collimation of 2 mm × 96 mm × 0.6 mm, a rotation time of 250 ms, tube voltage of 120 kV, and tube current of 350 − 650 mA. The standard scanning protocol was used during this study. When patients had a heart rate < 80/min and ≥ 80/min, prospective high-pitch spiral scanning mode (65% of relative risk interval) and sequence scanning mode (30-70% of relative risk interval) were performed, respectively. In total, 898 patients underwent prospective high-pitch spiral scanning mode with a radiation dose (dose length product, DLP) of 43.7±13.0 mGycm and 271 patients underwent sequence scanning mode with a radiation dose (DLP) of 303.1±135.0 mGycm. Volume rendering and curve planner reformation were performed using SIEMENS Syngo. A postprocessing workstation (SIEMENS).

### Plaque characteristics and FFR_CT_ analyses

CCTA coronary plaques were assessed in all vessels with diameters ≥ 2 mm. Plaques were defined as structures with areas ≥ 1 mm^2^ within and/or adjacent to the vessel lumen and clearly distinguished from the artery lumen and surrounding pericardial tissue.

All FFR_CT_ analyses were performed using the Frontier Scientific Research Platform postprocessing workstation (SIEMENS, Munich, Germany) as described, which has a good agreement with fractional flow reserve from the pressure wire in multi-center studies [13]. A three-dimensional coronary tree model and report were acquired using FFR_CT_ analysis. Hemodynamically significant stenosis caused by a lesion with significant hemodynamic changes was defined as an FFR_CT_ value ≤ 0.80 at the per-patient level [14].

**Fig. 2:**
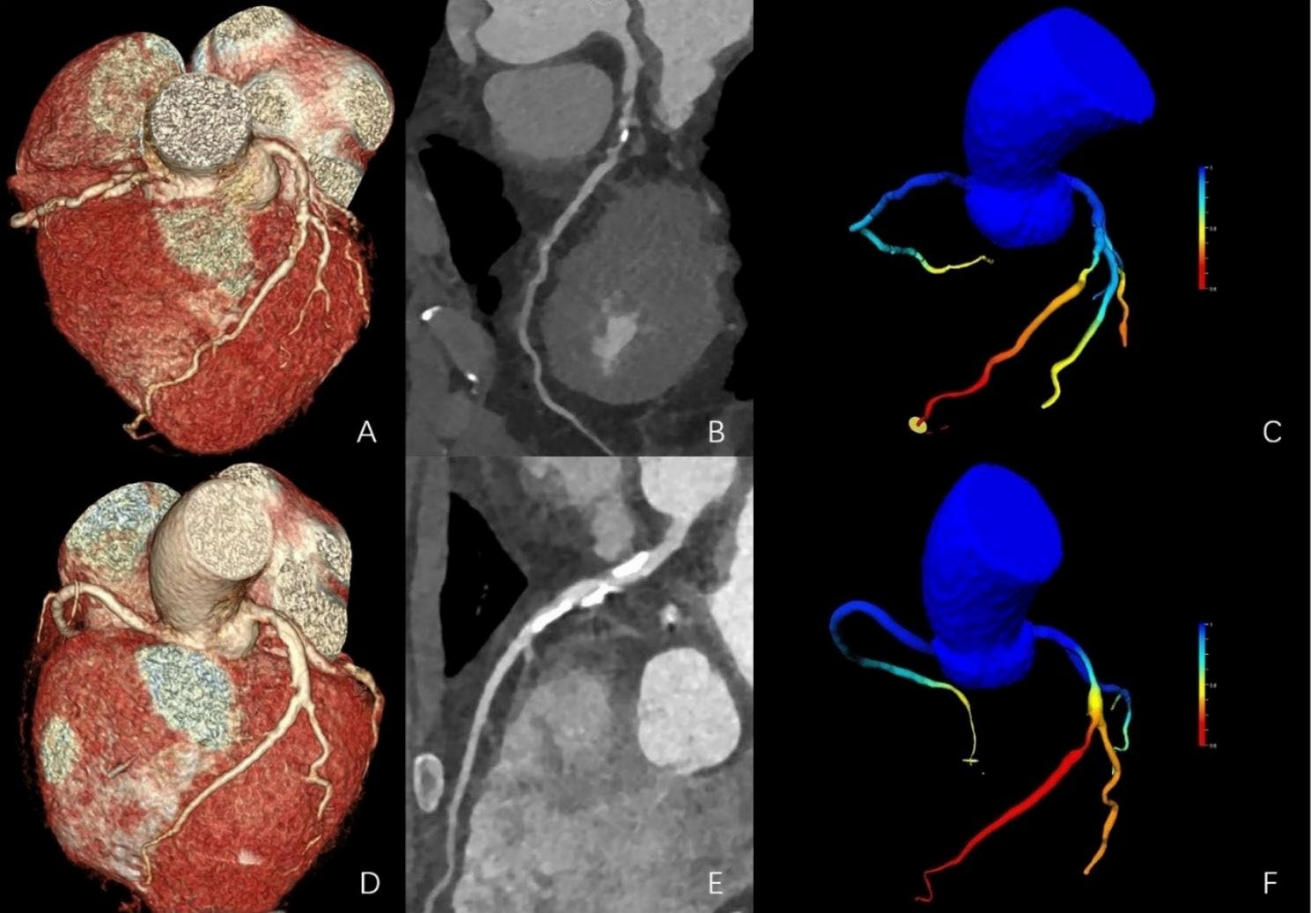
Representative case from the present study. CCTA showed severe (> 75%) stenosis in the proximal LAD with a mixture plaque of the non-calcified and calcified component and FFR_CT_ value of 0.75 in a 58-years old male with atypical chest pain (A-C). CCTA showed severe (> 75%) stenosis in proximal and middle LAD with a calcified plaque and FFR_CT_ value of 0.60 in a 55-years old female with atypical chest pain and chest tightness (D-F). Volume rending (A, D), LAD curve planner reformation (B, E), and FFR_CT_ analyses (C, F). CCTA = coronary computed tomography angiography, LAD = left anterior descending artery, FFR_CT_ = computed tomography–derived fractional flow reserve.

Plaque volume was calculated by subtracting the lumen volume from the total vessel volume at the per-patient level, including calcified plaques and non-calcified plaques (evaluated and reported as mm^3^) [15–17]. The percent total aggregated plaque volume (TAPV) was further calculated. The percent TAPV calculation formula was percent TAPV = [TAPV (mm^3^) / total vessel volume (mm^3^) × 100] [17]. All plaque volumes were standardized by the individual body surface area (BSA). The BSA calculation formula was BSA (m^2^) = [0.0061 × height (cm) + 0.0128 × body mass (kg) – 0.1529]. The non-calcified plaque volumes consisting of lipid-rich and fibrotic plaques were expressed as plaque volume with attenuation density < 30 HU and plaque volume with attenuation density 30 to 130 HU, respectively. The calcified plaque volume was defined as plaque volume with attenuation density > 130 HU. The subtype plaque volumes as non-normally distributed parameters were evaluated as qualitative dichotomous variables using the highest quartile threshold at the per-patient level. The proportions of subtype plaque were expressed as the ratios of the lipid-rich plaque volume (%), fibrotic plaque volume (%), and calcified plaque volume (%) to the total plaque volume. A case example is shown in Fig. 2.

### Statistical analysis

All statistical analyses were performed using SPSS Statistics version 26.0 (IBM Corporation, Armonk, NY, USA) and Stata software version 15.0 (StataCorp, College Station, TX, USA). For descriptive analysis, continuous variables are represented as the mean±standard deviation or median (interquartile range) as appropriate; categorical variables are expressed as numbers and percentages. Categorical and continuous variables were compared using the χ2 test, t-test, or Mann-Whitney U test. A regression analysis was performed with FFR_CT_ ≤0.80 or the plaque component highest quartile as a dichotomous outcome. The odds ratios [OR] and 95% confidence intervals [CI] were calculated using a multivariate binary logistic regression analysis for each risk factor with adjustments for confounding factors, including age, BMI, hypertension, diabetes mellitus, hyperlipidemia, and smoking status. The association between cardiovascular risk factors and percent TAPV was assessed using multivariate linear regression analyses and reported as a correlation coefficient (β). The discriminating capacity of diverse plaque components for hemodynamically significant stenosis was assessed by area under the receiver-operating characteristics curve with 95% confidence intervals. Statistical significance was defined as a two-sided P < 0.05. Intra-class correlation coefficients were calculated to evaluate inter- and intra-observer reproducibility. The calculation of sample size and statistical power was performed using GPower version 3.1 (Heinrich Heine University, Westphalia, Germany).

## Results

### Clinical characteristics

This study enrolled 1169 patients with clinically suspected CAD who underwent CCTA for plaque composition assessment and FFR_CT_ analyses. The 531 patients with atherosclerotic plaque were divided into the following two groups: 263 (49.5%) men and 268 (50.5%) women. Sex differences in the clinical characteristics are presented in Table 1. Women were older and more often had hyperlipidemia than men. A higher BMI, quitting smoking for ≥ 1 year or current smoking, and lower exercise frequency were the more prevalent characteristics for men than for women. The statistical power of the Chi-square and the Mann − Whitney U tests was 0.999 and 0.921, respectively.

**Table 1:** Clinical characteristics and imaging findings in men and women

	Men (N = 263)	Women (N = 268)	P value
Demographics			
Age (years)	54 (61–66)	63 (59–66)	< 0.001
BMI (kg/m^2^)	25.46 (23.51–27.62)	24.65 (22.63–26.91)	0.044
Exercise			0.007
no	97 (36.9%)	132 (49.3%)	
< 3 times per week	42 (16.0%)	26 (9.7%)	
≥ 3 times per week	124 (47.1%)	110 (41.0%)	
Traditional CAD risk factor			
Hypertension	144 (54.8%)	136 (50.7%)	0.355
Antihypertensive treatment (n, %)	131 (50.2%)	130 (49.8%)	0.125
Disease duration (years)	8 (4–12)	8 (3–14)	0.452
Diabetes mellitus	53 (20.2%)	50 (18.7%)	0.663
Hypoglycemia treatment (n, %)	47 (88.7%)	47 (94.0%)	0.339
Disease duration (years)	8 (3–12)	10 (4–12)	0.383
Hyperlipidemia	131 (49.8%)	160 (59.7%)	0.022
Lipid-lowing treatment (n, %)	38 (29.0%)	55 (34.4%)	0.329
Cerebrovascular disease	25 (9.5%)	22 (8.2%)	0.599
History of premature CAD disease	111 (42.4%)	122 (45.5%)	0.441
Cigarette smoking			< 0.001
Never	87 (33.1%)	256 (95.5%)	
Quitting smoking for ≥ 1 year	50 (19.0%)	3 (1.1%)	
Current smoker	126 (47.9%)	9 (3.4%)	
CCTA findings			
Quantitative stenotic grade	54.06±24.69	43.7±20.9	< 0.001
Patients with FFR_CT_≤0.8	175 (35.7%)	82 (12.1%)	< 0.001
The total number of atherosclerotic plaques	4 (2,9)	3 (1,5)	< 0.001
Percent TAPV	57.54 (42.31–68.32)	47.76 (31.65–59.84)	< 0.001
Calcified plaque volume (mm^3^/m^2^)	42.80 (15.66–133.00)	28.21 (11.25–79.35)	< 0.001
Lipid-rich plaque volume (mm^3^/m^2^)	2.83 (0.21–11.76)	0.25 (0.00-2.66)	< 0.001
fibrotic plaque volume (mm^3^/m^2^)	31.28 (10.13–73.52)	8.62 (0.85–30.62)	< 0.001
Plaque percentage (%)			
Calcified	65.40 (41.30-86.27)	87.16 (67.61–93.25)	< 0.001
Lipid-rich	2.25 (0.27–6.34)	0.41 (0.00-2.85)	< 0.001
fibrotic	28.59 (13.50-45.62)	12.45 (6.41–28.81)	< 0.001

P < 0.05 was considered statistically significant, and data was presented as mean ± SD, number (percentage) or median (interquartile range), BMI = body mass index, CAD = coronary artery disease, CCTA = coronary computed tomography angiography, FFR_CT_=computed tomography–derived fractional flow reserve, TAPV = percent total aggregated plaque volume

### Angiographic characteristics

Regarding hemodynamically significant stenosis identified by the FFR_CT_ ≤0.8, men had a higher percent TAPV, total number of atherosclerotic plaques, and prevalence of hemodynamically significant stenosis than women (Table 1). There was a significant sex difference in the subtype plaque composition in all patients (Table 1). Plaque composition analyses showed that each subtype plaque volume (calcified, fibrotic, and lipid-rich) was significantly higher in men than in women (all P < 0.001). Notably, the ratio of the subtype plaque volume to the total plaque volume differed across sexes; women had a higher percentage of calcified components, whereas men had a higher percentage of lipid-rich and fibrotic components (all P < 0.001). The statistical power of the t-test was 0.932. The inter- and intra-observer reproducibility for plaque components and hemodynamically stenosis were excellent (Additional File: Supplementary Material, Table S1).

### Associations between cardiovascular risk factors and plaque components

**Fig. 3:**
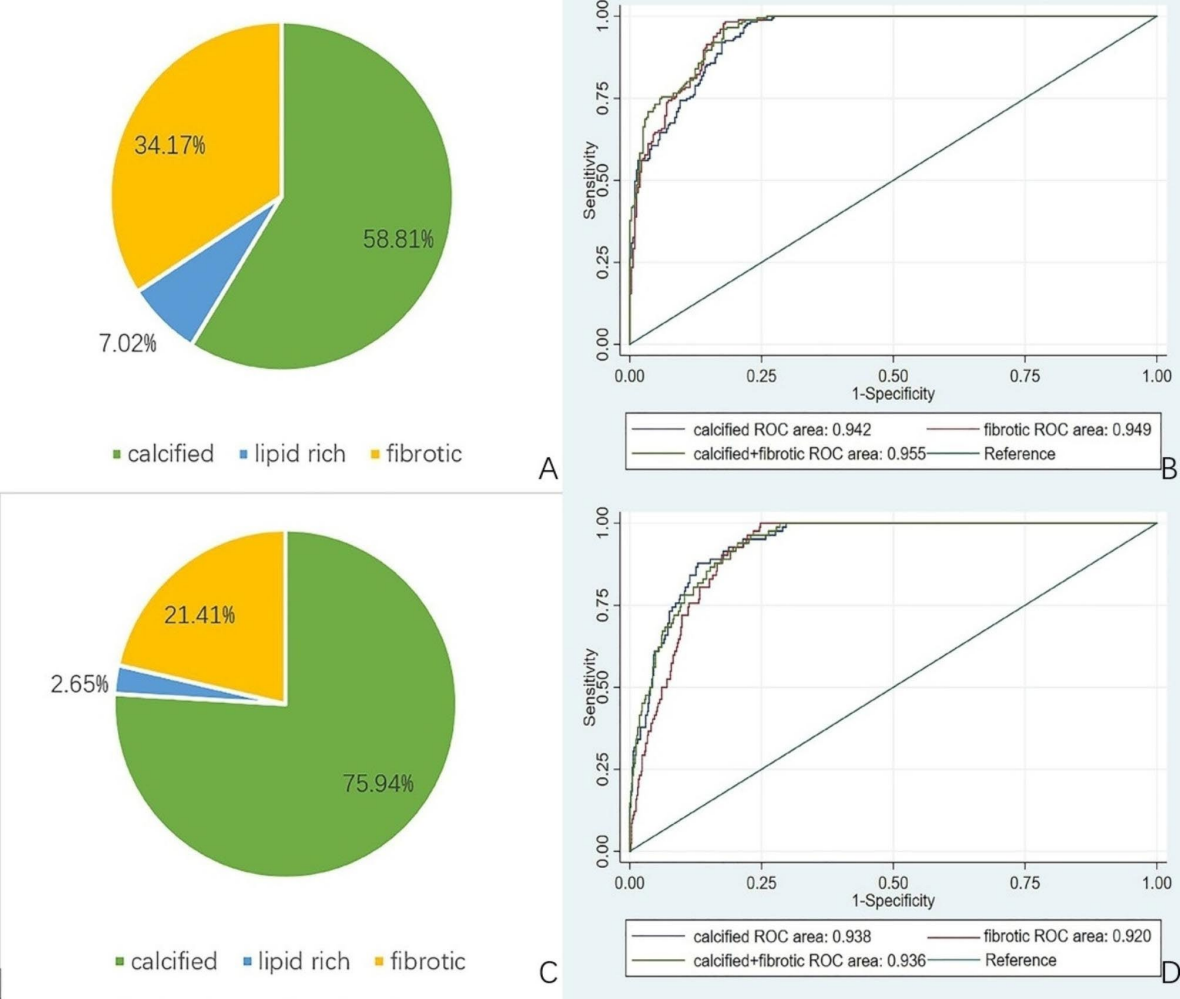
Association with coronary plaque composition and hemodynamically stenosis identified by FFR_CT_≤0.8 between males and females. In males with hemodynamically significant stenosis, the plaque volume was comprised of 58.81% calcified plaque volume, 34.17% fibrotic plaque volume and 7.02% lipid rich plaque volume (A). In females with hemodynamically significant stenosis, the plaque volume was comprised of 75.94% calcified plaque volume, 21.41% fibrotic plaque volume and 2.65% lipid rich plaque volume (C). AUCs for discrimination of hemodynamically significant stenosis in men (B) and in women (D). FFR_CT_ = computed tomography–derived fractional flow reserve, AUC = area under the receiver-operating characteristics curve.

There was a significant sex difference in the association between cardiovascular risk factors and plaque components (Tables 2 and 3). For men, hyperlipidemia and cigarette smoking were risk factors for each plaque component (all P < 0.05). Diabetes mellitus (P = 0.025) was a risk factor for fibrotic components (Table 2). The prediction of the subtype plaque composition highlighted the significance of hypertension and diabetes for women compared to that for men (all P < 0.01). There was a slight difference in the predictions of calcified and non-calcified components in women; hyperlipidemia (P < 0.05) was the risk factor for the non-calcified component rather than the calcified component (Table 3). There were significant associations among the duration of illness, medication use, and plaque components in women rather than in men. In women, the duration of diabetes mellitus (OR: 1.134; 95% CI: 1.035 − 1.242; (P = 0.007) and lipid-lowering therapy (OR: 1.601; 95%CI: 1.045 − 2.453; P = 0.031) were risk factors for lipid-rich components. Furthermore, the duration of hypertension was a risk factor for calcified components (OR: 1.036; 95% CI: 1.004 − 1.070; P = 0.028). In patients with hyperlipidemia, the calcified component volume increased significantly when receiving lipid-lowering therapy (P = 0.025) (Additional File: Supplementary Material, Figure S1). A plaque composition analysis revealed that the most abundant plaques in individuals with hemodynamically significant stenosis were the calcified components, followed by fibrotic and lipid-rich components both in men and women (Fig. 3A and B). For men, the discriminative capacity of calcified components for hemodynamically significant stenosis was the same as that of fibrotic components (P = 0.310). The addition of fibrotic components to calcified components provided an incremental prediction of hemodynamically significant stenosis (P = 0.004); however, the discriminative capacity of hemodynamically significant stenosis of fibrotic components was not improved further by the addition of calcified components (P = 0.118). For women, the discriminative capacity of calcified components for hemodynamically significant stenosis was stronger than that of fibrotic components (P = 0.026). The addition of calcified components to fibrotic components provided an incremental prediction of hemodynamically significant stenosis (P = 0.011). Nonetheless, the discriminative capacity of hemodynamically significant stenosis of calcified components was not improved further by the addition of fibrotic components (P = 0.408) (Fig. 3C, D **and** Table 4). The statistical power of the regression analysis was 0.919.

**Table 2:** Binary multivariate logistic regression for sub-type plaque composition (HQ) in men

	Calcified		Lipid-rich		Fibrotic	
	OR (95% CI)	*P* value	OR (95% CI)	*P* value	OR (95% CI)	*P* value
Age (years)	1.123 (1.089–1.158)	< 0.001	1.074 (1.047–1.101)	< 0.001	1.078 (1.051–1.107)	< 0.001
BMI	0.999 (0.922–1.081)	0.975	1.068 (0.994–1.147)	0.073	1.045 (0.971–1.124)	0.243
Hypertension	1.202 (0.752–1.923)	0.442	1.109 (0.710–1.733)	0.649	1.167 (0.743–1.833)	0.503
Diabetes mellitus	1.184 (0.650–2.156)	0.581	1.531 (0.871–2.689)	0.139	1.901 (1.085–3.330)	0.025
Hyperlipidemia	2.160 (1.342–3.475)	0.002	1.611 (1.032–2.514)	0.036	1.767 (1.125–2.776)	0.013
Cigarette smoking	1.499 (1.141–1.969)	0.004	1.380 (1.070–1.781)	0.013	1.446 (1.114–1.876)	0.006

P < 0.05 was considered statistically significant. CI = confidence interval, OR = odds ratio

**Table 3:** Binary multivariate logistic regression for sub-type plaque composition (HQ) in women

	Calcified		Lipid-rich		Fibrotic	
	OR (95% CI)	*P* value	OR (95% CI)	*P* value	OR (95% CI)	*P* value
Age (years)	1.097 (1.065–1.130)	< 0.001	1.072 (1.043–1.103)	< 0.001	1.076 (1.046–1.107)	< 0.001
BMI	0.990 (0.940–1.042)	0.695	1.020 (0.969–1.073)	0.457	0.997 (0.947–1.049)	0.904
Hypertension	1.836 (1.260–2.676)	0.002	1.794 (1.230–2.616)	0.002	1.932 (1.326–2.814)	0.001
Diabetes mellitus	2.082 (1.267–3.421)	0.004	2.037 (1.244–3.337)	0.005	2.519 (1.546–4.105)	< 0.001
Hyperlipidemia	1.437 (0.984–2.099)	0.060	1.772 (1.208–2.599)	0.003	1.581 (1.082–2.310)	0.018
Cigarette smoking	0.993 (0.596–1.654)	0.978	1.256 (0.794–1.988)	0.330	1.257 (0.794–1.989)	0.330

P < 0.05 was considered statistically significant. CI = confidence interval, OR = odds ratio

**Table 4:** Discriminative capacity of hemodynamically significant stenosis of each component in men and women

	Calcified	Fibrotic	Calcified and Fibrotic	*P* value	*P*_calcified_ _vs. fibrotic_	*P*_calcified vs._ _calcified+fibrotic_	*P*_fibrotic vs._ _calcified+fibrotic_
	AUC (95% CI)	AUC (95% CI)	AUC (95% CI)				
Men	0.942 (0.923–0.960)	0.949 (0.932–0.966)	0.955 (0.940–0.970)	< 0.001	0.310	0.004	0.118
Women	0.938 (0.918–0.957)	0.920 (0.899–0.941)	0.936 (0.916–0.956)	0.024	0.026	0.408	0.011

P < 0.05 was considered statistically significant. CI = confidence interval, AUC = area under the receiver-operating characteristics curve

### Associations among cardiovascular risk factors, percent TAPV, and hemodynamically significant stenosis

**Fig. 4:**
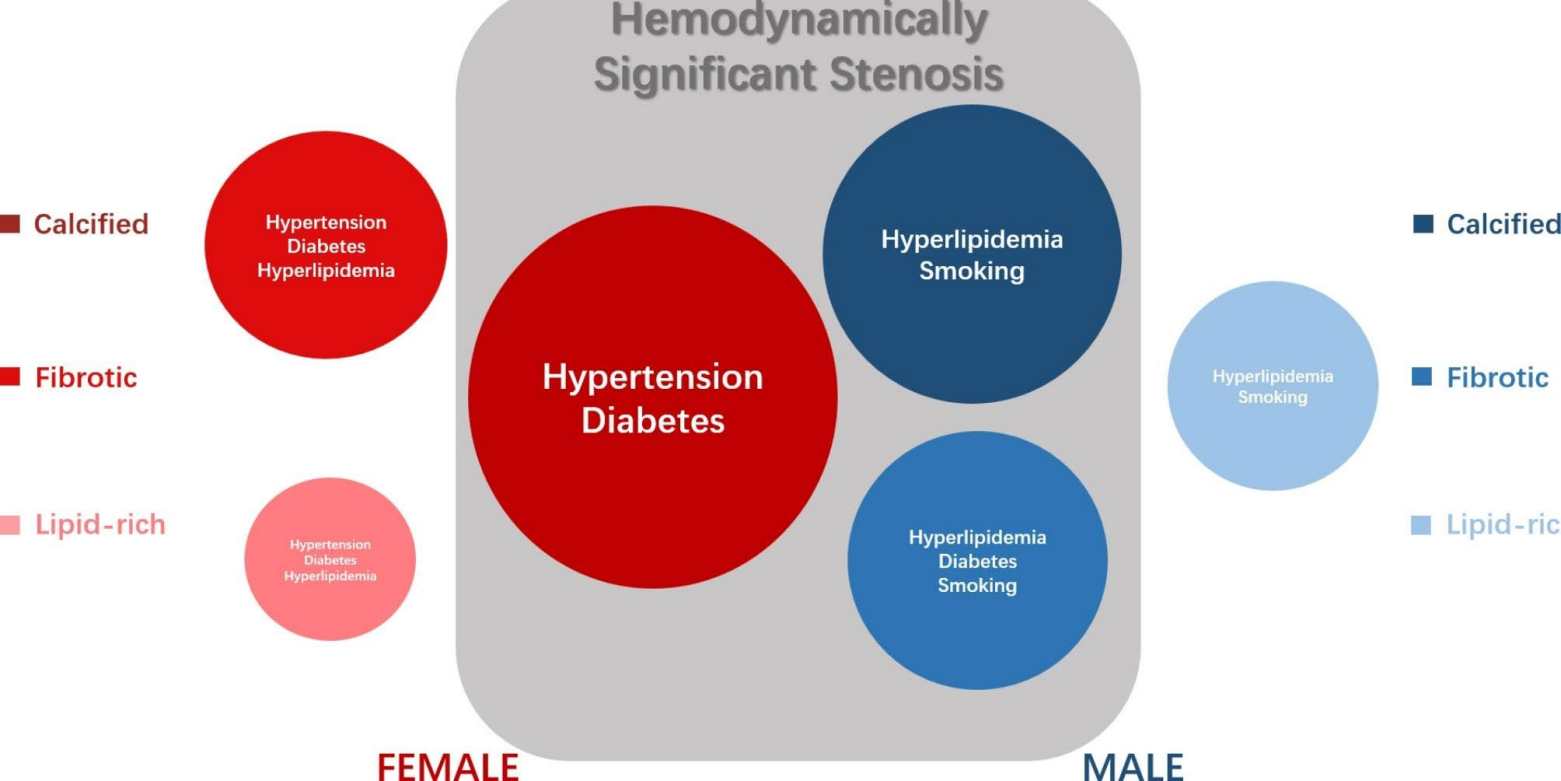
Sex difference in the associations with risk factor, coronary plaque composition and hemodynamically significant stenosis

According to multivariate regression analysis performed at the per-patient level, there were sex differences in the cardiovascular risk factors for predicting hemodynamically significant stenosis and *percent TAPV* (Tables 5 and 6). Hypertension (P < 0.01), which was not observed in men, was a risk factor for hemodynamically significant stenosis and *percent TAPV* in women; furthermore, diabetes (P < 0.001) was a risk factor for *percent TAPV* in women. The risk factors of hemodynamically significant stenosis were diabetes mellitus (P = 0.038), hyperlipidemia (P = 0.011), and cigarette smoking (P = 0.002) in men, which were consistent with the risk factors for *percent TAPV*. There were significant differences in risk factors of the corresponding components for predicting hemodynamically significant stenosis among men and women (Fig. 4).

**Table 5:** Binary multivariate logistic regression for hemodynamically significant stenosis identified by FFR_CT_

	Men		Women	
	OR (95% CI)	*P* value	OR (95% CI)	*P* value
Age (years)	1.095 (1.069–1.122)	< 0.001	1.106 (1.062–1.153)	< 0.001
BMI	1.028 (0.961-1.100)	0.423	1.004 (0.941–1.072)	0.898
Hypertension	1.056 (0.695–1.605)	0.798	1.994 (1.218–3.264)	0.006
Diabetes mellitus	1.801 (1.033–3.139)	0.038	1.677 (0.906–3.105)	0.100
Hyperlipidemia	1.723 (1.131–2.623)	0.011	1.120 (0.868–1.828)	0.650
Cigarette smoking	1.463 (1.152–1.857)	0.002	1.279 (0.703–2.329)	0.421

P < 0.05 was considered statistically significant. CI = confidence interval, OR = odds ratio

**Table 6:** Multivariate linear regression for percent TAPV

	Men		Women	
	β (SE)	P value	β (SE)	P value
Age (years)	1.027 (0.376)	< 0.001	0.772 (0.246)	< 0.001
Hypertension	-	-	5.729 (0.110)	0.003
Diabetes mellitus	8.830 (0.105)	0.013	10.056 (0.132)	< 0.001
Hyperlipidemia	5.024 (0.083)	0.047	-	-
Cigarette smoking	3.144 (0.094)	0.024	-	-

P < 0.05 is considered statistically significant. β = unstandardized coefficient, SE = standard error, TAPV = percent total aggregated plaque volume

## Discussion

Patients with suspected CAD underwent CCTA with plaque composition assessment and FFR_CT_ analyses. The results showed that the relationships among cardiovascular risk factors, subtype plaque components, and hemodynamically significant stenosis differed between men and women.

### Sex differences in plaque composition and hemodynamically significant stenosis

Compared to the findings of a previous study, men had a significantly higher incidence of hemodynamically significant stenosis and more severe coronary atherosclerosis than did women, regardless of the plaque components [18]. Furthermore, Lee et al. found that women had greater calcified plaque volume progression but slower non-calcified plaque volume (lipid-rich and fibrotic plaques) progression than did men, which may lead to a higher proportion of calcified components and a lower proportion of non-calcified components in women [18].

### Associations between cardiovascular risk factors and plaque components

Regarding the significance of the subtype plaque component, associations between cardiovascular risk factors and subtype plaque components varied more in men but approximately identical in women. Diabetes was significantly associated with each subtype component in women but with only the fibrotic component in men. It is worth noting that diabetes significantly increased the lipid-rich components volume in women, although it was not related to hemodynamically significant stenosis. Especially, the longer duration of diabetes furtherly promoted the development of lipid-rich components in women. Therefore, diabetes maybe a more important risk factor for lipid-rich components in women than in men, which is a possible explanation for why women with diabetes are at a relatively higher risk for fatal CAD or major adverse cardiovascular events induced by the rupture of plaque with a large necrotic core [19–22]. In summary, strict glycemic control in women is crucial to prevent high-risk plaque rupture and corresponding acute coronary syndrome.

Compared with women, men had hyperlipidemia that not only significantly increased the volume of non-calcified components as a risk factor for a worse prognosis, which was elucidated by a previous pathological study that found a ruptured plaque with large lipid cores covered by a thin fibrous cap formed from dead and dying cells with lipoprotein particles [7, 21], but also significantly facilitated calcified component progression. In other words, men with hyperlipidemia experienced volume progression of each subtype and had increased total plaque volume that developed into obstructive CAD. These results could explain why men with myocardial infarction were more likely to have hyperlipidemia than women [23]. Nevertheless, women receiving lipid-lowing therapy were significantly associated with lipid-rich components. Furthermore, lipid-lowering therapy has been shown to be associated with increased volumes of calcified components, which is consistent with our findings. In the future, the developments and changes in subtype plaque components should be monitored to analyze sex differences in the prognoses of cases involving hyperlipidemia.

In women, hypertension exhibited a stronger positive relationship with each subtype of plaque component than it did in men. This result may be attributed to smaller vessels and more severe arterial stiffness caused by hypertension in women than in men [24, 25]. Furthermore, Torngren et al. found that arterial stiffness was associated with a high coronary artery calcification score and had mechanisms similar to those of endothelial dysfunction in atherosclerotic development, which might explain the longer duration of hypertension as a risk factor for calcified components in women [26]. Hence, hypertension is a higher attributable risk factor for myocardial infarction in women than in men [27].

The present study found that cigarette smoking was significantly associated with each subtype of plaque components in men. It also found that, compared to diabetes and hypertension, cigarette smoking was one of the most significant predictors of lipid-rich components, which is in agreement with the results of a previous study [28]. Therefore, quitting cigarette smoking is an efficient way for men to prevent major adverse cardiovascular events. There was no sex difference in the associations with age, plaque composition, and the incidence of hemodynamically significant stenosis in the present study.

### Associations among cardiovascular risk factors, percent TAPV, and hemodynamically significant stenosis

By analyzing further risk factors for percent TAPV and hemodynamically significant stenosis, it was found that hypertension was the risk factor for percent TAPV and hemodynamically significant stenosis in women, while diabetes, hyperlipidemia, and cigarette smoking were risk factors for percent TAPV and hemodynamically significant stenosis in men.

In men with hemodynamically significant stenosis, the present study found that the diagnostic ability for hemodynamically significant stenosis discrimination was significantly improved by adding calcified and fibrotic components than by using calcified components only. Furthermore, the discriminative capacity of calcified components for hemodynamically significant stenosis was the same as that of fibrotic components. Therefore, we have reasons to believe that the combination of calcified and fibrotic components might have a dominating diagnostic ability for hemodynamically significant stenosis in men. On the other hand, in women with hemodynamically significant stenosis, the calcified component was the major coronary plaque component causing hemodynamically significant stenosis.

As mentioned above, hypertension was a risk factor of hemodynamically significant stenosis predicted by calcified components in women, while diabetes, hyperlipidemia, and cigarette smoking were risk factors of hemodynamically significant stenosis predicted by calcified components combined with fibrotic components in men. Notably, the present study demonstrated that diabetes was a risk factor for calcified components and percent TAPV, and the association with diabetes and hemodynamically significant stenosis in women was validated in univariate regression but not in multivariate regression analysis. This difference may be because FFR_CT_ simulates the ratio of the mean coronary pressure distal to a coronary stenosis to the mean aortic pressure to determine hemodynamically significant stenosis by a mathematical model. In the setting of microvascular dysfunction, elevated pressure distal to a critical stenosis may result in a normal pressure drop across a hemodynamically significant lesion [29]. Furthermore, Haas et al. also found that women with diabetes had worse myocardial perfusion and diastolic function than men, leading to more extensive coronary microvascular dysfunction [30]. Therefore, we conclude that diabetes facilitated the progression of calcified components, which might lead to hemodynamically significant stenosis; however, it is necessary to further verify whether diabetes was associated with hemodynamically significant stenosis in a larger sample size of female patients.

The traditional Diamond and Forrester model with age-based, sex-based, and symptom-based pretest probabilities has been used to estimate the presence of obstructive coronary artery disease (CAD ≥ 50%) in autopsy [31]. During the past decade, with the development of CCTA, pretest probabilities combined with other CCTA results (such as epicardial fat volume) were found to be more accurate and effective, substantially reducing the overestimations observed using the Diamond and Forrester model [32]. Further sex stratifications based on associations with cardiovascular risk factors, plaque composition, and hemodynamically significant stenosis could result in enriched and improved pretest probability inter-relations.

### Limitations

This study had several limitations. First, CT was limited in differentiating different types of non-calcified plaque, which was a significant overlap between fibrotic and more lipid-rich plaque and thrombus, and there was always a significant bias in plaque volume measured using different software. Second, this was a single-center and observational study, which could result in bias. Finally, the sex differences in the smoking status were presented, which might have influenced the impact of smoking on CAD among women because fewer women were smokers in this study.

## Conclusions

There were significant sex differences in the associations among cardiovascular risk factors, plaque compositions, and hemodynamically significant stenosis. In men, the association was more varied and complicated; diabetes, hyperlipidemia, and cigarette smoking as predictors of calcified components combined with fibrotic components were risk factors for hemodynamically significant stenosis. On the other hand, in women, the association was approximately consistent and straightforward. Hypertension, as one of the calcified component predictors, was a risk factor for hemodynamically significant stenosis. The diverse plaque components exhibited distinct implications for the progression of hemodynamically significant stenosis. These differences suggest the need for sex-specific prevention of hemodynamically significant stenosis with corresponding plaque composition. With the development of CCTA and artificial intelligence, the inaccuracy of the CT threshold may be further overcome, and artificial intelligence may reduce the manual measurement bias of different software to make the quantitative analysis of plaque more accurate.

### Electronic supplementary material

Below is the link to the electronic supplementary material.

Additional File: Supplementary Material, Table S1 and figure S1

## Data Availability

The datasets presented in this article are not readily available because containing information that could compromise the privacy of participants. Please contact the corresponding author for data requests. Requests to access the datasets should be directed to Dong Li, dr_lidong@163.com.

## Abbreviations

CAD: Coronary artery disease
CCTA: Coronary computed tomography angiography
FFR_CT_: Computed tomography–derived fractional flow reserve
BMI: Body mass index
BSA: Body surface area
