# Supplementary material for: Sex-specific associations of cardiovascular risk factors and coronary plaque composition for hemodynamically significant coronary artery stenosis: a coronary computed tomography angiography study
Source: BMC Cardiovasc Disord. 2023 Aug 27;23:423. doi: 10.1186/s12872-023-03438-x (PMC10463363; doi:10.1186/s12872-023-03438-x)
Supplement: Supplementary file 1 — Additional File: Supplementary Material, Table S1 and figure S1 [file 12872_2023_3438_MOESM1_ESM.docx]

**Supplementary Material Table S1** Inter- and intra-observer reproducibility analysis

|  | Intra-observer | | Inter-observer | |
| --- | --- | --- | --- | --- |
|  | ICC | 95%CI | ICC | 95%CI |
| Calcified | 0.940 | 0.791-0.974 | 0.897 | 0.764-0.946 |
| Lipid-rich | 0.953 | 0.925-0.969 | 0.887 | 0.799-0.932 |
| Fibrotic | 0.955 | 0.936-0.969 | 0.921 | 0.887-0.944 |
| FFR_CT_ | 0.934 | 0.907-0.954 | 0.830 | 0.764-0.878 |

ICC=Intra-class correlation coefficient, CI= confidence interval

**Supplementary Material Figure S1** Calcified component volume between the lipid-lowering therapy and the non-lipid-lowering therapy groups


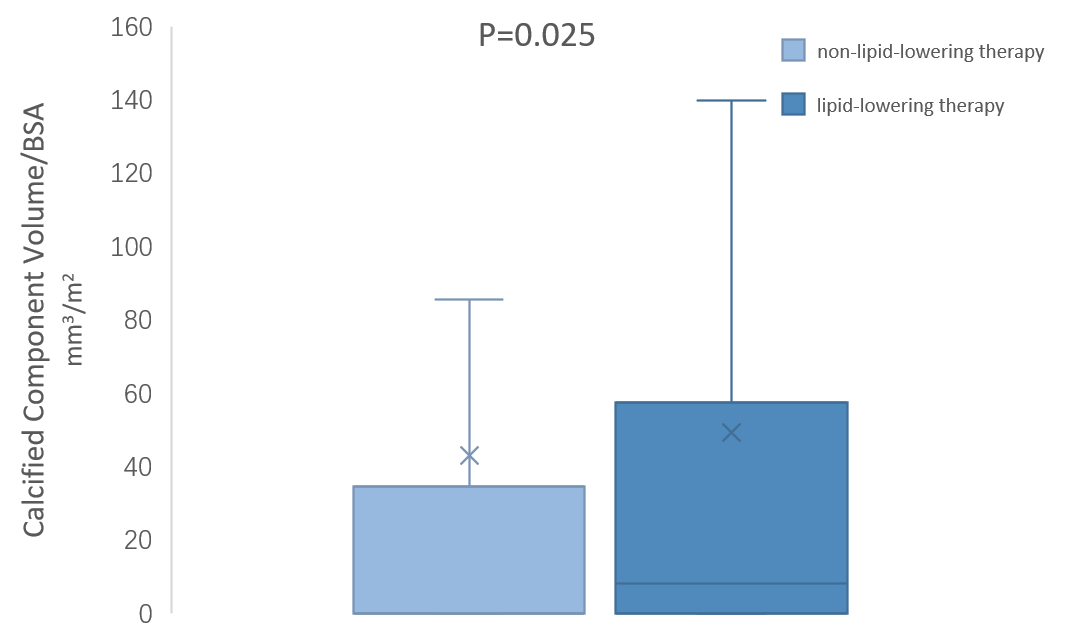


BSA= body surface area.
